# Supplementary figures and images for: No behavioural evidence for rhythmic facilitation of perceptual discrimination
Source: Eur J Neurosci. 2021 May 4;55(11-12):3352–64. doi: 10.1111/ejn.15208 (PMC9540985; doi:10.1111/ejn.15208)

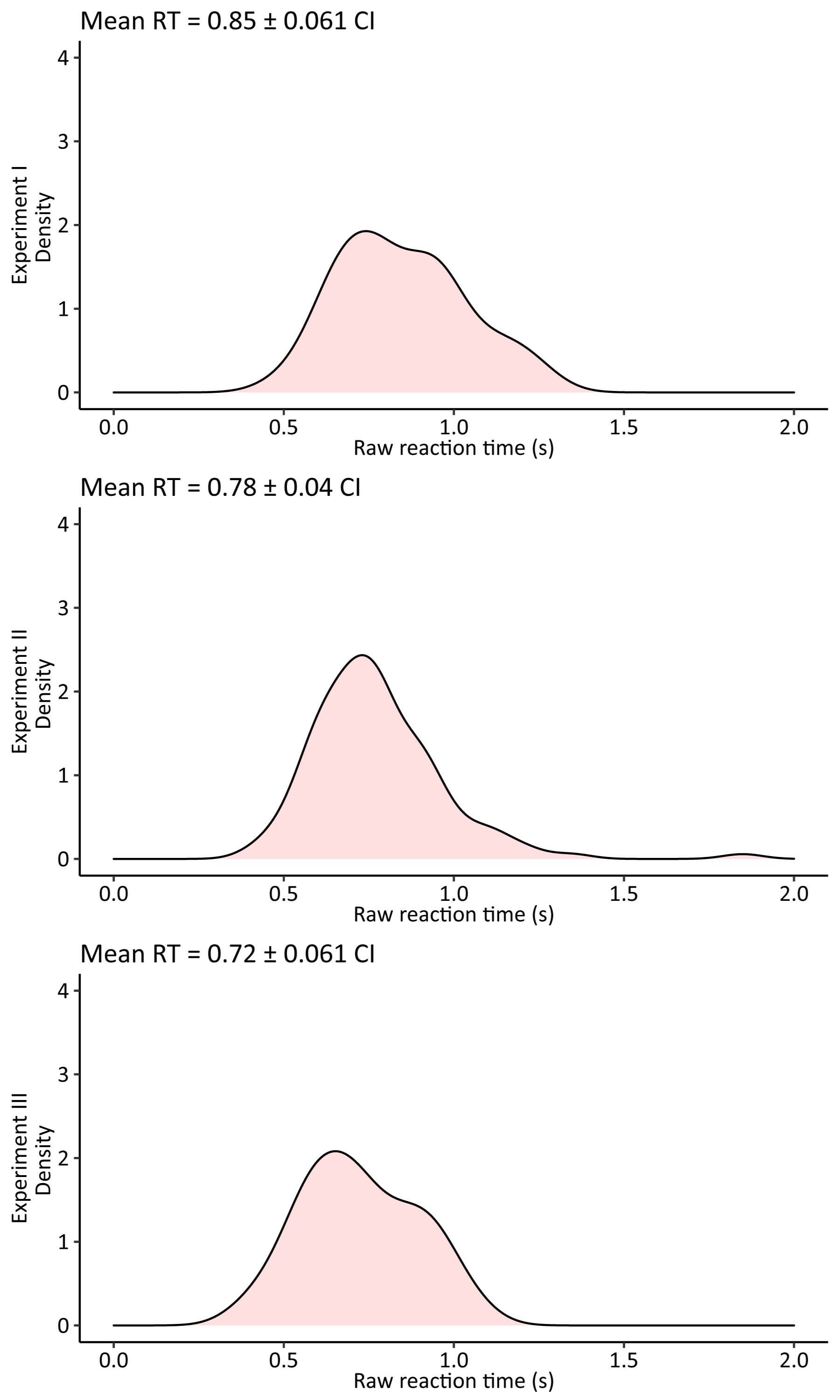

Supplement: Supplementary file 1 — Fig S1 [file EJN-55-3352-s001.tif]

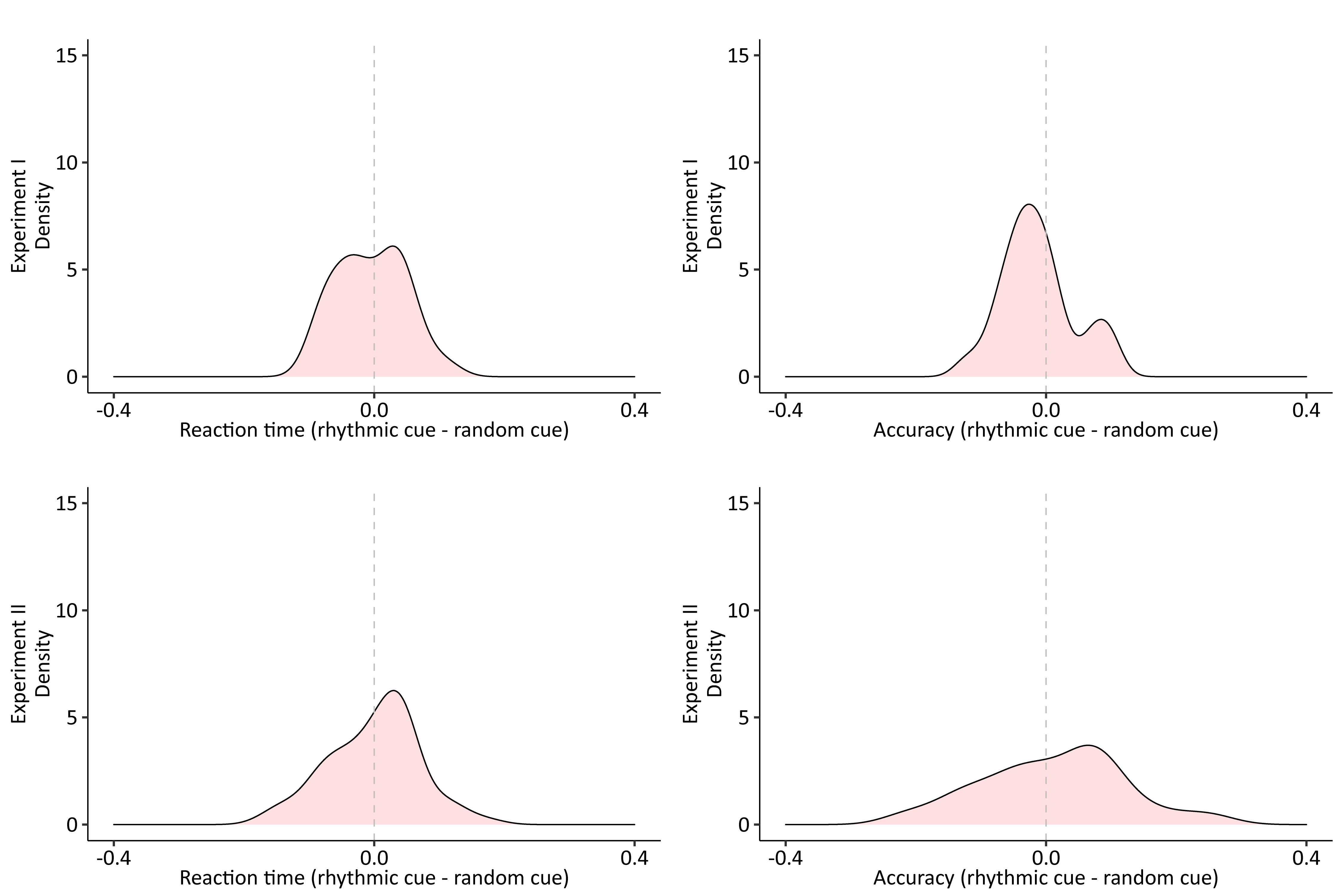

Supplement: Supplementary file 2 — Fig S2 [file EJN-55-3352-s003.tif]

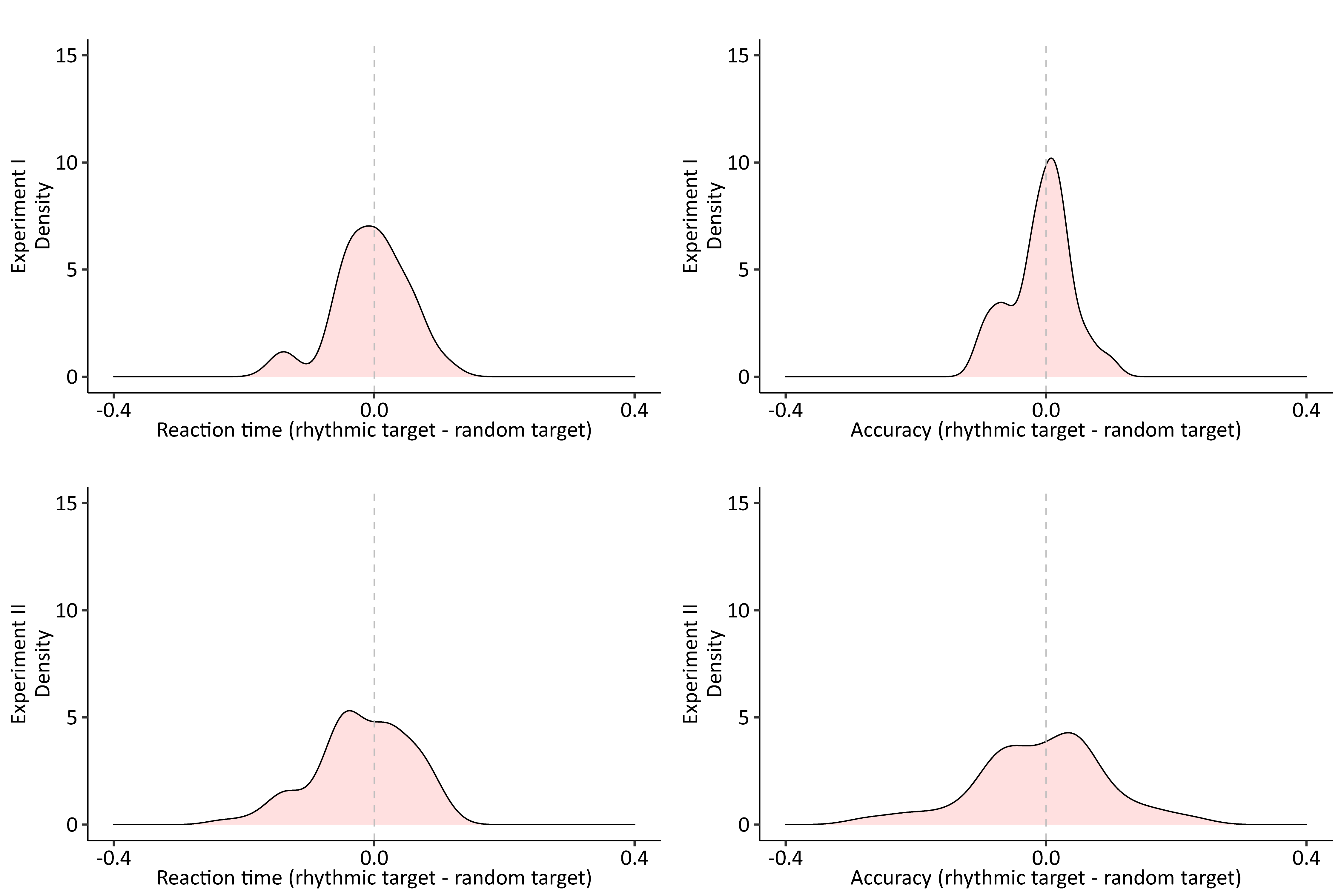

Supplement: Supplementary file 3 — Fig S3 [file EJN-55-3352-s005.tif]

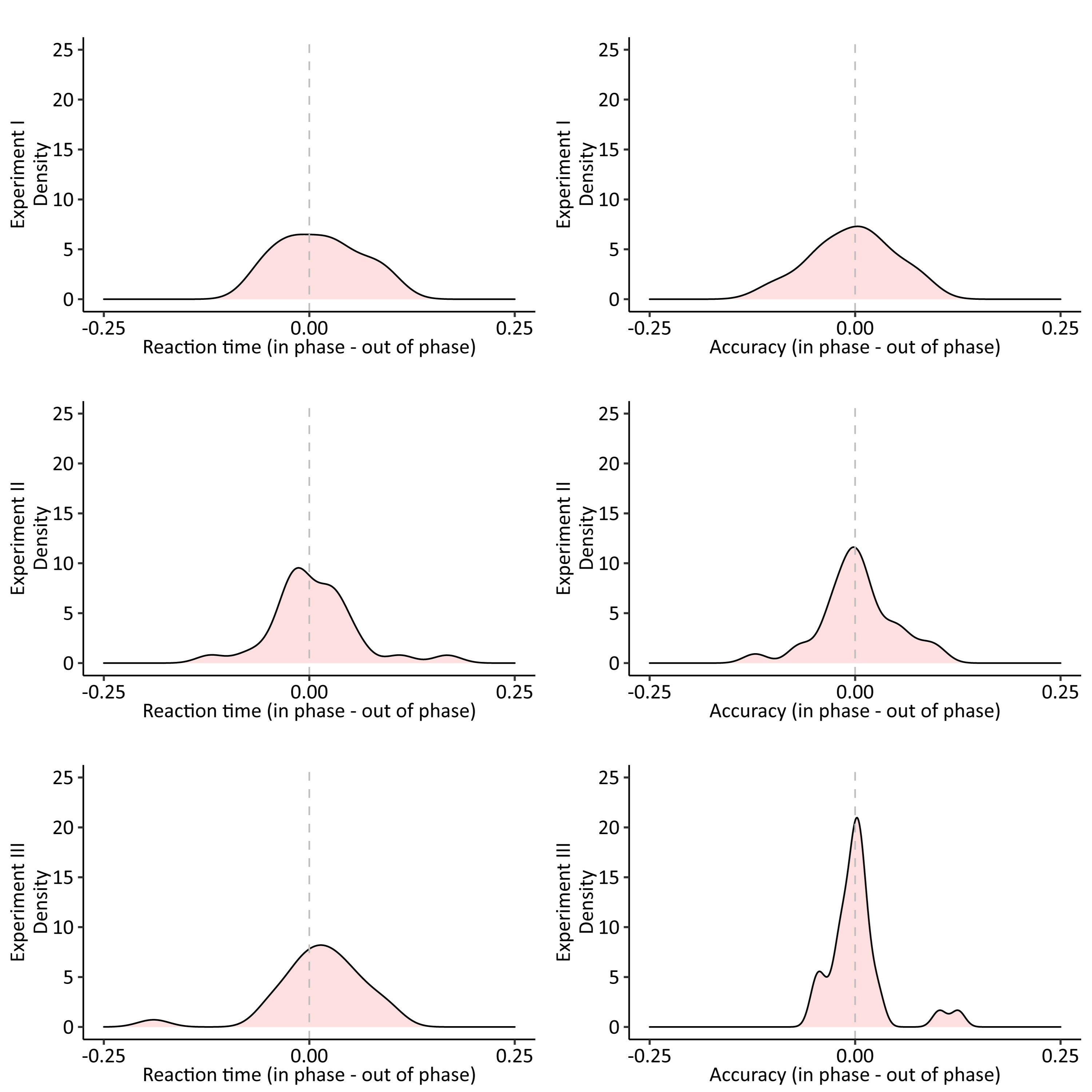

Supplement: Supplementary file 4 — Fig S4 [file EJN-55-3352-s002.tif]
