# Supplementary material for: No behavioural evidence for rhythmic facilitation of perceptual discrimination
Source: Eur J Neurosci. 2021 May 4;55(11-12):3352–64. doi: 10.1111/ejn.15208 (PMC9540985; doi:10.1111/ejn.15208)
Supplement: Supplementary file 5 — Supplementary Material [file EJN-55-3352-s004.docx]

# Supplementary figures


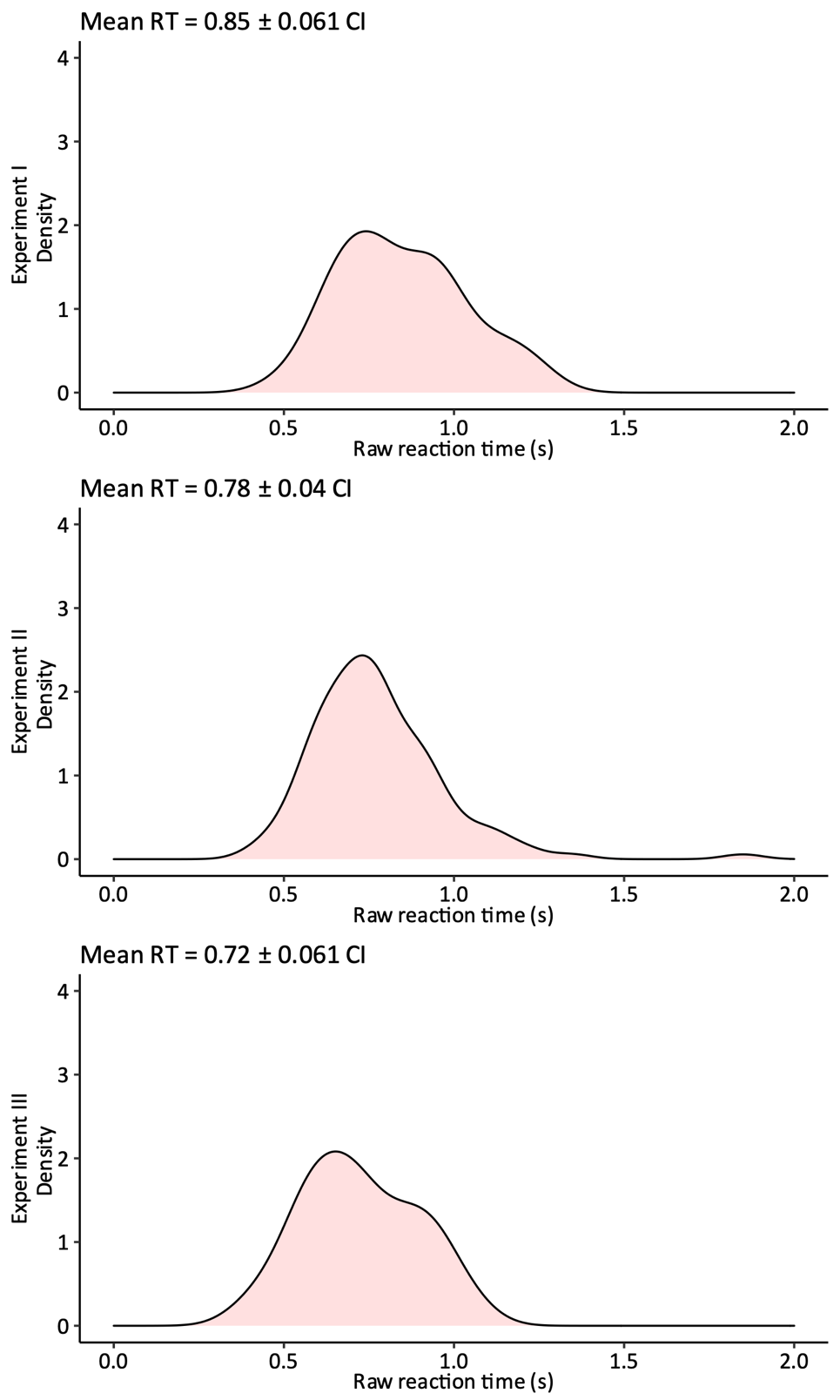


**Supp. fig. 1: Raw reaction times.** Histograms of mean individual-level raw reaction times prior to normalization and outlier exclusion, for experiments I (top), II (middle), and III (bottom).


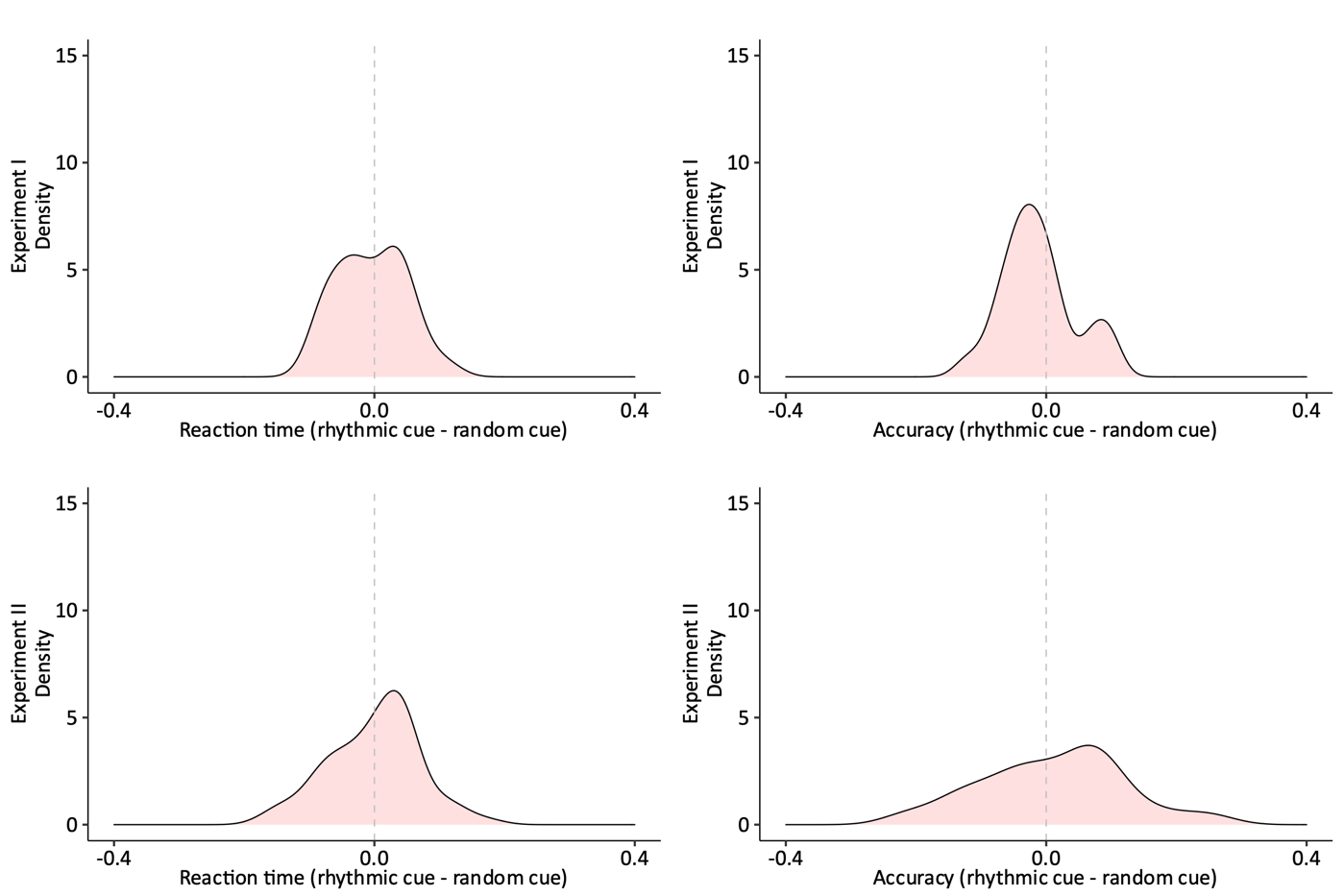


**Supp. fig. 2: Cue rhythmicity effects**. Histograms of mean individual-level reaction time (left) and accuracy (right) differences between rhythmic and random cue conditions for experiments I (top) and II (bottom).


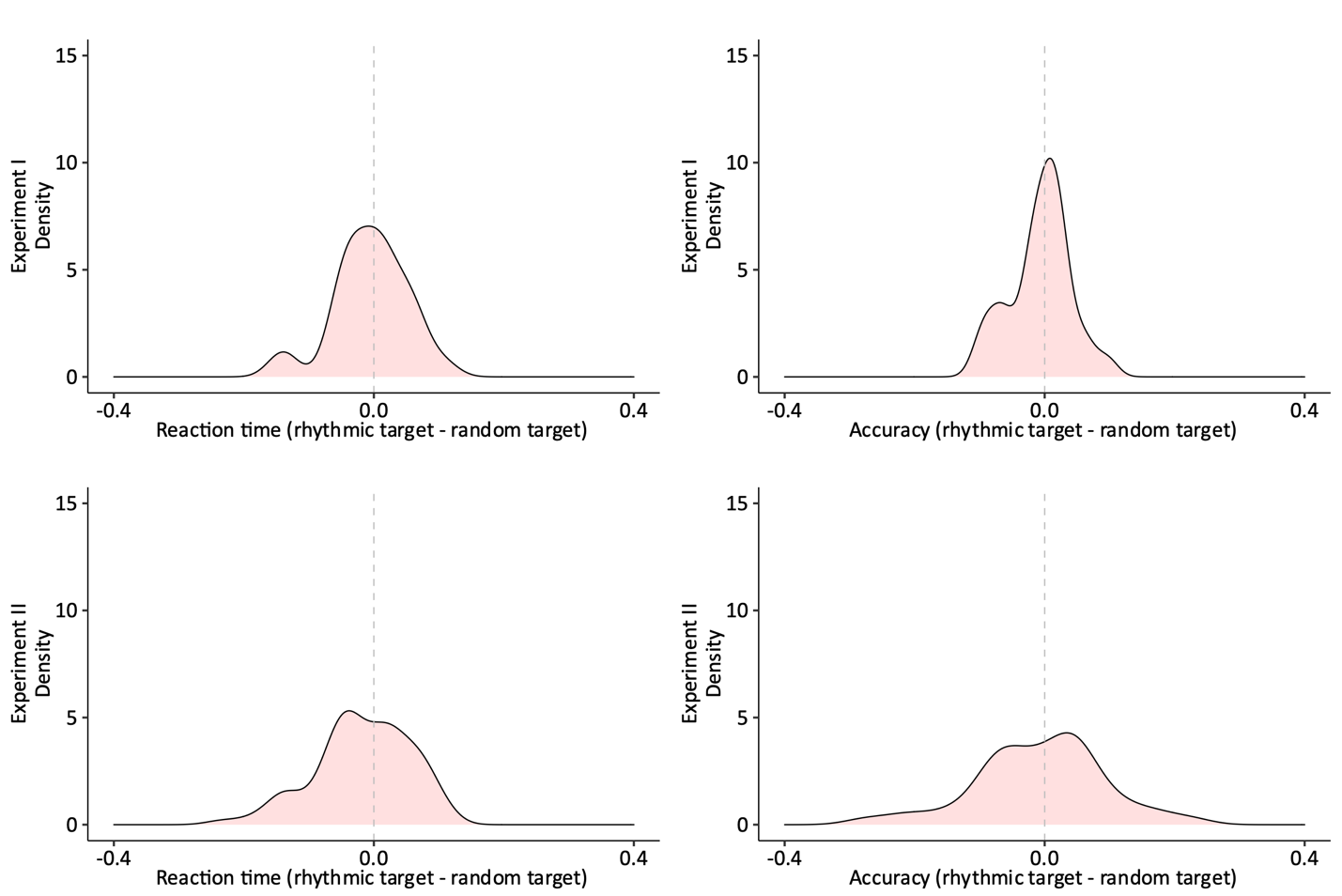


**Supp. fig. 3: Target rhythmicity effects**. Histograms of mean individual-level reaction time (left) and accuracy (right) differences between rhythmic and random target conditions for experiments I (top) and II (bottom).

**
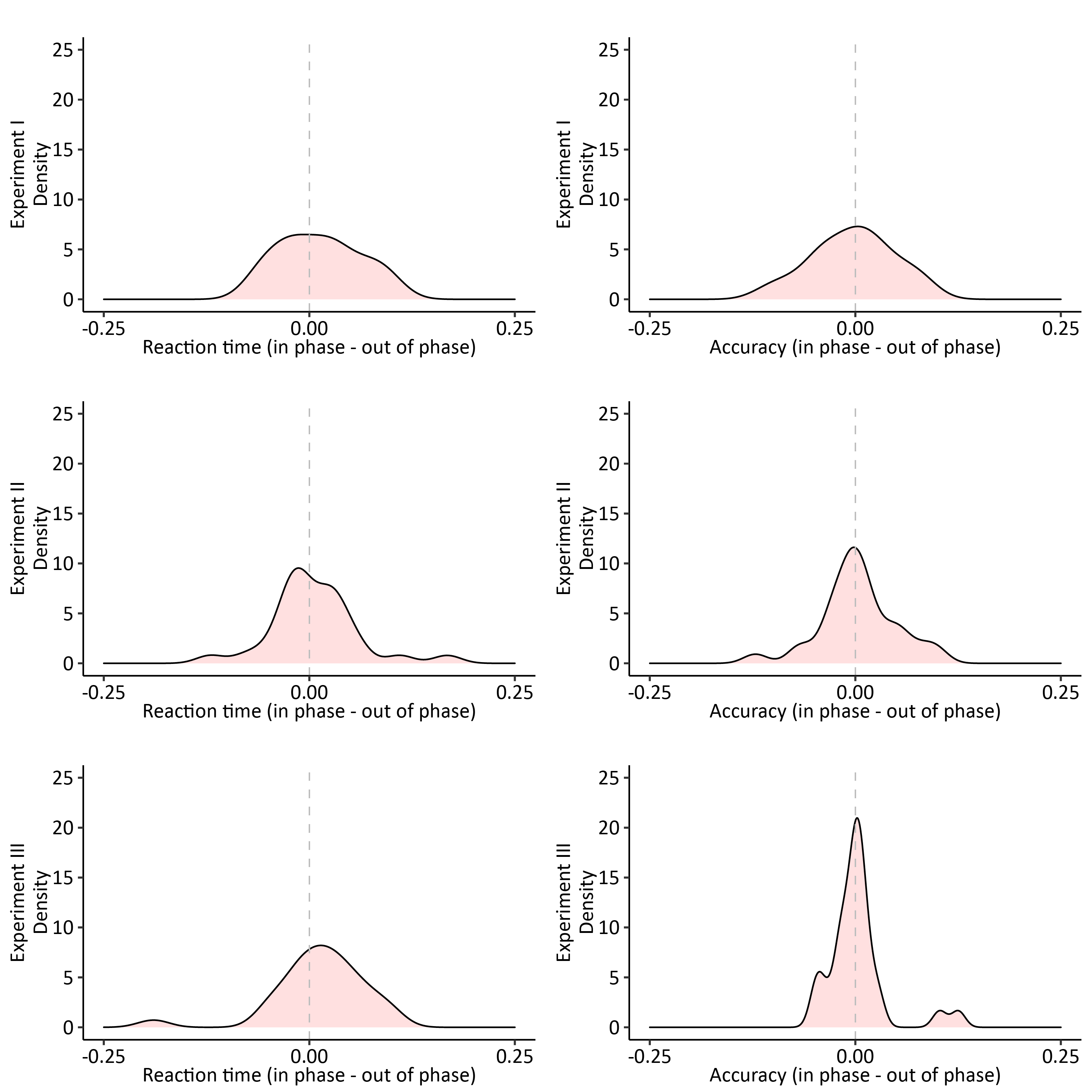
**

**Supp. fig. 4: Phase effects.** Histograms of mean individual-level reaction time (left) and accuracy (right) differences between in-phase and out-of-phase target conditions for experiments I (top), II (middle), and III (bottom).

# Supplementary tables

| Test | Experiment | Accuracy | | | Reaction time | | |
| --- | --- | --- | --- | --- | --- | --- | --- |
|  |  | mean | min | max | mean | min | max |
| cue ✻ target ✻ period | I | 45 | 8* | 52 | 38 | 7* | 49 |
|  | II | 154 | 120 | 179 | 128 | 64 | 174 |
| period | III | 45 | 38 | 48 | 38 | 23 | 48 |
| cue ✻ phase | I | 35 | 29 | 36 | 29 | 19 | 35 |
|  | II | 105 | 95 | 108 | 84 | 56 | 102 |
| phase | III | 117 | 110 | 120 | 98 | 63 | 117 |

**Supp. table 1:** Mean, minimum, and maximum number of trials used for all tests reported in the main manuscript, calculated for each combination of levels of the ANOVA factors. *Only one participant had this minimum number of trials, with the next lowest having 36 (accuracy) and 19 (reaction time) trials.

| **Cases** | **Accuracy** | | | **Reaction time** | | |
| --- | --- | --- | --- | --- | --- | --- |
|  | **F-value** | **p-value** | **BF_10_** | **F-value** | **p-value** | **BF_10_** |
| cue | 2.656 | .114 | 0.20 | 0.858 | .362 | 0.05 |
| target | 0.715 | .405 | 0.06 | 0.223 | .640 | 0.05 |
| period | 0.095 | .909 | 0.01 | 15.093 | < .001^gg^ | 12.87 |
| cue ✻ target | 0.029 | .866 | 0.018 | 0.192 | .664 | 0.01 |
| cue ✻ period | 1.646 | .202 | 0.007 | 3.049 | .059^gg^ | 0.034 |
| target ✻ period | 0.175 | .840 | < 0.001 | 0.182 | .825^gg^ | 0.02 |

**Supp. table 2:** Results of the repeated-measures ANOVA (**experiment I**) with factors cue rhythmicity (rhythmic, random), target rhythmicity (rhythmic, random), and period (0.5, 0.6, and 0.7) on accuracy and reaction time after removing the modelled hazard rate effect. Tests to which Greenhouse-Geisser correction was applied as the assumption of sphericity was violated are indicated with gg.

| **Cases** | **Accuracy** | | | **Reaction time** | | |
| --- | --- | --- | --- | --- | --- | --- |
|  | **F-value** | **p-value** | **BF_10_** | **F-value** | **p-value** | **BF_10_** |
| cue | 1.483 | .226 | 0.08 | 0.010 | .920 | 0.23 |
| target | 0.061 | .805 | 0.06 | 2.333 | .130 | 0.18 |
| period | 6.579 | .002 | 31.03 | 80.421 | < .001 | > 100 |
| cue ✻ target | 0.637 | .426 | 0.01 | 3.122 | .080 | 0.14 |
| cue ✻ period | 1.599 | .204 | 0.12 | 5.162 | .20 | 0.47 |
| target ✻ period | 0.224 | .799 | 0.06 | 1.140 | .322 | 0.12 |

**Supp. table 3:** Results of the repeated-measures ANOVA (**experiment II**) with factors cue rhythmicity (rhythmic, random), target rhythmicity (rhythmic, random), and period (0.4, 0.6, and 0.9) on accuracy and reaction time after removing the modelled hazard rate effect.

| **Cases** | **Accuracy** | | | **Reaction time** | | |
| --- | --- | --- | --- | --- | --- | --- |
|  | **F-value** | **p-value** | **BF_10_** | **F-value** | **p-value** | **BF_10_** |
| period | 0.83 | .58 | 0.02 | 28.14 | < .001* | > 100 |

**Supp. table 4:** Results of the repeated-measures ANOVA (**experiment III**) with factor period (10 levels) on accuracy and reaction time after removing the modelled hazard rate effect. Asterisk signifies test to which Greenhouse-Geisser correction was applied as the assumption of sphericity was violated.

| **Cases** | **Accuracy** | | | **Reaction time** | | |
| --- | --- | --- | --- | --- | --- | --- |
|  | **F-value** | **p-value** | **BF_10_** | **F-value** | **p-value** | **BF_10_** |
| cue | 2.4 | .13 | 0.94 | 1.39 | .24 | 0.4 |
| phase | 0.11 | .74 | 0.17 | 2.98 | .1 | 0.29 |
| cue ✻ phase | 1.82 | .18 | 0.17 | 0.09 | .75 | 0.11 |

**Supp. table 5:** Results of the repeated-measures ANOVA (**experiment I**) with factors cue rhythmicity (rhythmic, random) and target phase (in, out) on accuracy and reaction time after removing the modelled hazard rate effect.

| **Cases** | **Accuracy** | | | **Reaction time** | | |
| --- | --- | --- | --- | --- | --- | --- |
|  | **F-value** | **p-value** | **BF_10_** | **F-value** | **p-value** | **BF_10_** |
| cue | 0.004 | .94 | 0.14 | 0.53 | .46 | 0.32 |
| phase | 0.03 | .85 | 0.15 | 1.81 | .18 | 0.36 |
| cue ✻ phase | 0.12 | .72 | 0.03 | 0.1 | .76 | 0.12 |

**Supp. table 6:** Results of the repeated-measures ANOVA (**experiment II**) with factors cue rhythmicity (rhythmic, random) and target phase (in, out) on accuracy and reaction time after removing the modelled hazard rate effect.

|  | **Accuracy** | | | **Reaction time** | | |
| --- | --- | --- | --- | --- | --- | --- |
| **df** | **t-value** | **p-value** | **BF_10_** | **t-value** | **p-value** | **BF_10_** |
| 28 | 0.35 | .72 | 0.21 | 1.97 | .06 | 1.06 |

**Supp. table 7:** Results of the t-test (**experiment III**) contrasting accuracy and reaction times for target in and out of phase after removing the modelled hazard rate effect.
